# A Novel *MAP3K7* Variant Causing Loss of Function Identified in a Family With Cardiospondylocarpofacial Syndrome: Functional Validation and Molecular Insights

**DOI:** 10.1155/humu/8024677

**Published:** 2026-04-24

**Authors:** Ting Zhu, Jiamin Shi, Jia Li, Tingmin Zhou, Xinru Fu, Fengzhen Xu, Chuangjie Gu, Danping Wang, Ruiting Wu, Li Liu, Jingling Shen, Dan Wang

**Affiliations:** ^1^ Department of Anesthesia and Surgery, Wenzhou Central Hospital of Wenzhou Medical University, Wenzhou, Zhejiang Province, China; ^2^ Department of Pediatrics, The First Affiliated Hospital of Wenzhou Medical University, Wenzhou, Zhejiang, China, wzhospital.cn; ^3^ Ultrasound Department, Shaoxing People′s Hospital, Shaoxing, Zhejiang, China; ^4^ Department of Neurology, The First Affiliated Hospital of Wenzhou Medical University, Wenzhou, Zhejiang, China, wzhospital.cn; ^5^ Department of Pediatrics, Taizhou Hospital of Zhejiang Province Affiliated to Wenzhou Medical University, Taizhou, Zhejiang, China, wmu.edu.cn; ^6^ Institute of Life Sciences, College of Life and Environmental Sciences, Wenzhou University, Wenzhou, Zhejiang, China, wzu.edu.cn; ^7^ Consortium for Infection and Innovation (CII), The First Affiliated Hospital of Wenzhou Medical University, Wenzhou, Zhejiang, China, wzhospital.cn

**Keywords:** cardiospondylocarpofacial syndrome, frontometaphyseal dysplasia type 2, *MAP3K7*, TAK1, TGF-*β*

## Abstract

Mitogen‐activated protein kinase kinase kinase 7 (*MAP3K7*), also known as transforming growth factor‐*β*–activated kinase 1 (TAK1), is a widely expressed kinase that plays a crucial role in various cellular processes variants in the *MAP3K7* gene have been implicated in two distinct genetic disorders: frontometaphyseal dysplasia Type 2 (FMD2) and cardiofaciocutaneous syndrome (CSCF). To elucidate the consequences of the *MAP3K7* variant, we investigated a Chinese family with CSCF harboring a novel heterozygous *MAP3K7* variant and examined the genotype–phenotype correlation. Functional validation was performed using clinical evaluations, whole‐exome sequencing (WES), and biochemical assays, including western blotting to assess TAK1 phosphorylation levels and downstream signaling pathways. Clinical data and genomic DNA were collected from the proband and family members. WES identified a novel heterozygous variant in *MAP3K7* (NM_145331.3: c.149 T > C, p.Val50Ala) inherited from the affected mother. Sequence conservation analysis revealed that the Val50 residue is highly conserved among vertebrates and is critical for ATP binding. Protein 3D modeling predicted that the Val50Ala variant disrupts the kinase domain structure, potentially impairing TAK1 function. In vitro overexpression experiments in human embryonic kidney 293T (HEK293T) cells demonstrated that the Val50Ala variant significantly reduced TAK1 phosphorylation levels. Furthermore, this variant differentially affected downstream signaling molecules (p38, p65, and JNK) compared with variants causing FMD2. Notably, stimulation with transforming growth factor‐*β* (TGF‐*β*) partially restored the altered phosphorylation patterns, suggesting a potential compensatory mechanism. Our study provides novel insights into the molecular pathogenesis of *MAP3K7* variants associated with CSCF and FMD2. We demonstrate that the p.Val50Ala variant impairs TAK1 kinase activity and differentially affects downstream signaling pathways. These findings highlight the distinct molecular fingerprints of *MAP3K7* variants causing CSCF versus FMD2 and underscore the importance of considering *MAP3K7* variants in the differential diagnosis of syndromic congenital cardiac defects, recurrent infections, and global developmental delays. Our results also suggest that TGF‐*β* signaling may offer a potential therapeutic target for modulating the effects of *MAP3K7* variants.

## 1. Introduction

*MAP3K7* (OMIM#602614), also known as transforming growth factor β‐activated kinase 1 (*TAK1*), as a member of the serine/threonine protein kinase family, can be activated by various cytokines and is involved in numerous physiological functions, including growth, differentiation, inflammation, and apoptosis [1, 2]. TAK1 can phosphorylate a series of target proteins, thereby activating distinct signaling pathways and cellular responses under varying stress conditions or in different cell types. The most common TAK1‐mediated signaling pathways include the MAPK kinase 3/6‐p38 MAPK pathway, the nuclear p65 pathway, and the c‐Jun N‐terminal kinase (JNK) pathway [3–5]. Variants in *MAP3K7* are associated with two distinct autosomal dominant disorders: loss‐of‐function cardiospondylocarpofacial (CSCF) syndrome (OMIM#157800) and gain‐of‐function frontometaphyseal dysplasia Type 2 (FMD2) (OMIM#617137) [6].

CSCF syndrome is characterized by growth retardation, dysmorphic facial features, brachydactyly with carpal–tarsal fusion and extensive posterior cervical vertebral synostosis, cardiac septal defects with valve dysplasia, and deafness with inner ear malformations [7]. FMD2 is a progressive skeletal disorder characterized by supraorbital hyperostosis, undermodeling of small bones, joint contractures, extraskeletal abnormalities, hearing loss, cardiac malformations, stenosis of the upper airway and urinary tract, and keloid scars [8]. To date, 48 individuals from 45 independent families with pathogenic (P) or likely pathogenic (LP) *MAP3K7* variants have been described (File S1). As the number of cases increases, significant overlap in the clinical manifestations of these two diseases has been observed Table 1.

**Table 1:** Summary of pathogenic *MAP3K7* variants and the clinical presentation of CSCF and FMD2.

	Gender	Postnatal measurements	Weeks of gestation (weeks)	Weight, Gr (SDS)	Length, cm (SDS)	Head circumference,cm (SDS)	
CSCF	F (17/25) M (8/25)		36–42 (23/23)	<−2 (5/23), Nor (18/23)	<−2 (2/17), Nor (15/17)	> +2 (2/14), Nor (12/14)	
FMD2	F (12/22) M (10/22)		34–35 (2/2)	<−2 (2/3), Nor (1/3)	Nor (2/2)	<−2 (1/2), Nor (1/2)	

	**Measurements Last Evaluation**	**Weight, kg (SDS)**	**Length, cm (SDS)**	**Head circumference, cm (SDS)**			
CSCF		<−2 (5/21), Nor (16/21)	<−2 (18/24), Nor (6/24)	Nor (19/19)			
FMD2		Nor (2/2)	<−2 (3/3)	<−2 (1/1)			

	**Facial**	**Low posterior hair line**	**Hypotonic face**	**Full cheeks**	**Low-set ears**	**Posteriorly rotated ears**	
CSCF		+(3/10), −(7/10)	+(6/14), −(8/14)	+(18/22), −(4/22)	+(9/15), −(6/15)	+(14/21), −(7/21)	
FMD2		−(2/2)	+(1/2), −(1/2)	+(3/3)	−(2/2)	−(2/2)	

	**Hypertelorism**	**Strabismus**	**Ptosis**	**Palpebral fissures**	**Epicanthal Folds**	**Peri/supra-orbital fullness**	
CSCF	+(18/22), −(4/22)	+(8/19), −(11/19)	+(12/17), −(5/17)	+(12/23), −(11/23)	+(9/14), −(5/14)	+(12/16), −(4/16)	
FMD2	+(22/22)	−(1/1)	−(1/1)	+(21/22), −(1/22)	+(2/2)	+(20/21), −(1/21)	

	**Anteverted nares**	**Round-tipped nose**	**Long philtrum**	**High-arched palate**	**Micrognathia**	**Cleft palate**	
CSCF	+(17/23), −(6/23)	+(17/18), −(1/18)	+(15/18), −(3/18)	+(6/9), −(3/9)	+(7/17), −(10/17)	+(1/1)	
FMD2	−(2/2)	+(3/4), −(1/4)	−(2/2)	+(1/1)	+(18/22), −(4/22)	+(6/18), −(12/18)	

	**Congenital stridor/subglottic stenosis**	**Dental malocclusion**	**Cardiac phenotype**	**Congenital heart defect**	**Septal defects**	**Cardiomyopathy**	**Valve dysplasia**
CSCF	U	+(3/3)		+(13/19), −(6/19)	+(4/18), −(14/18)	+(5/12), −(7/12)	+(13/13)
FMD2	+(6/15), −(9/15)	U		+(4/4)	+(2/2)	−(2/2)	+(2/2)

	**Skeletal Phenotype**	**Joint Laxity**	**Short extremities**	**Scoliosis**	**Vertebral fusion**		
CSCF		+(15/16), −(1/16)	+(7/7)	+(8/18), −(10/18)	+(9/9)		
FMD2		+(1/3), −(1/3)	U	+(16/19), −(3/19)	+(18/21), −(3/21)		

	**Flexion contractures**	**Flared metaphyses**	**Brachydactyly**	**Camptodactyly**	**Pectus excavatum**		
CSCF	+(1/9), −(8/9)	U	+(16/19), −(3/19)	+(1/13), −(12/13)	+(5/17), −(12/17)		
FMD2	+(21/21)	+(14/16), −(2/16)	+(1/2), −(1/2)	+(3/3)	−(1/1)		

	**Neurologic phenotype**		**Hypotonia**	**Muscle hypoplasia**	**Intellectual disability**	**Behavior disorders/development retardation**	**MRI brain**
CSCF			+(10/12), −(2/12)	+(3/5), ‐ (2/5)	+(1/16), −(15/16)	+(5/8). −(3/8)	+(3/6), −(3/6)
FMD2			+(1/2), −(1/2)	+(2/2)	+(5/21), −(16/21)	U	−(1/1)

	**Digestive System**	**Severe failure to thrive**	**Oro-pharyngeal difficulties**	**Gastro-esophageal reflux**	**Genitourinary System**	**Vesico-ureteral reflux**	**Hydronephrosis**
CSCF		+(8/8)	+(4/6), −(2/6)	+(5/6), −(1/6)		+(1/5), −(4/5)	U
FMD2		+(1/1)	U	U		+(1/1)	+(3/15), −(12/15)

	**Ear, nose, and throat**	**Recurrent otitis**	**Hearing Loss**	**Inner ear malformation**	**Cryptorchidism**	**Other**	**Keloid**
CSCF		+(6/6)	+(14/20), −(6/20)	+(6/6)	+(3/15), −(12/15)		U
FMD2		+(2/2)	+(17/19), −(2/19)	U	+(2/3), −(1/3)		+(9/19), −(10/19)

Abbreviations: Nor, normal; U, unknown.

**Figure 1:**
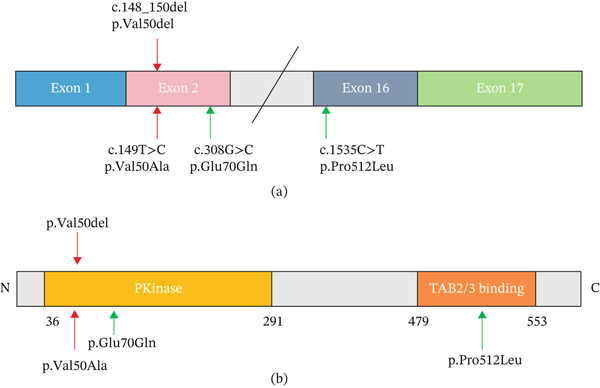
Localization of variation. The red arrow indicates the variant reported in the study. (a). Schematic representation of the MAP3K7 protein and location of the variants functionally validated in this study. Coding exons are numbered 1, 2, 16, and 17 (Exons 3–15 are omitted for simplicity). The green arrows indicate previously reported variants that were functionally validated in this study. (b). Key domains of the TAK1 protein. N‐terminal PKinase domain and the C‐terminal TAB2/3 binding domain. p.Val50 and p.Glu70 are located in N‐terminal PKinase domain. p.Pro512 is located in C‐terminal TAB2/3 binding domain.

TAK1 is a key regulator of inflammatory responses and is composed of two distinct domains: the N‐terminal kinase domain (amino ‐acids 36–291) and the C‐terminal TAB2/3 binding domain (amino acids 479–553) (Figure 1a,b) [9]. As a kinase, TAK1 relies on adenosine triphosphate (ATP) for its activity, with the ATP‐binding site located at Amino Acids 43–48 and 104–111. Upon ATP binding to its binding site, Thr178, Thr187, Thr184, and Ser192 within the activation loop of TAK1 are phosphorylated, leading to the activation of TAK1 [10]. The activated TAK1 molecule then phosphorylates downstream signaling molecules. Thus, the correct positioning of the ATP molecule is crucial for TAK1 activation.

In this study, we identified a novel loss‐of‐function *MAP3K7* missense variant, c.149 T > C (p.Val50Ala), in a Chinese family with CSCF and comprehensively investigated its clinical and genetic characteristics. We used bioinformatics tools and in vitro cellular functional verification to evaluate the pathogenicity of this variant and systematically elucidated genotype–phenotype correlations in patients with *MAP3K7* variants associated with both CSCF and FMD2. Additionally, for the first time, we utilized TGF‐*β* stimulation to compare downstream effects, thereby identifying potential new avenues for clinical treatment.

## 2. Materials and Methods

### 2.1. Participants

The proband and family members were enrolled at the First Affiliated Hospital of Wenzhou Medical University in May 2022. Written informed consent was obtained from the family of the proband before the beginning of the study. Our study was approved by the Ethics Committee of the First Affiliated Hospital of Wenzhou Medical University (Ethics Approval Number KY2022‐R177).

### 2.2. Whole‐Exome Sequencing (WES) and Sanger Sequencing

WES was conducted on the family. Genomic DNA was extracted from 3 to 5 mL of peripheral blood using the Qiagen DNeasy Blood & Tissue Kit (Qiagen, Hilden, Germany) following the manufacturer′s protocol. Exome enrichment was performed using an IDT xGen Exome Research Panel v2.0 Kit (Integrated DNA Technologies, Santa Clara, California, United States) following the manufacturer′s protocol. Then, the products were sequenced on a NovaSeq 6000 sequencer (Illumina, San Diego, California, United States). The quality of the raw sequencing data was assessed using fastp software [11], and low‐quality reads and adaptor‐contaminated reads were discarded. The filtered data were then aligned to the human hg19 reference genome using Burrows–Wheeler Aligner software. Subsequently, the capture efficacy was assessed. The Genome Analysis Toolkit was used to identify single‐nucleotide variants and insertions and deletions in the genome. The identified SNVs and indels from this family were then screened against the OMIM, HGMD, and ClinVar population databases. Based on the American College of Medical Genetics and Genomics (ACMG) guidelines for the evaluation of genetic variants [12], the variants were categorized as P, LP, variant of uncertain significance (VUS), likely benign (LB), or benign (B). Sanger sequencing was performed to verify suspected variants in the family [13].

### 2.3. Conservation Analysis and P Assessment

Sequence conservation of the TAK1 protein was examined using the ClustalW, SnapGene programs, and PhastCons. The potential pathogenicity of gene variants on protein function was assessed using the Franklin (https://franklin.genoox.com), SIFT (http://sift-dna.org), and MutationTaster (https://www.mutationtaster.org) websites (Table 2). Alphafold 3.0 (https://www.alphafoldserver.com) and Pymol were used to predict the three‐dimensional structure.

**Table 2:** Pathogenicity predictions of the variant.

Gene	Chromosome	Nucleotide	Amino acid	Franklin	SIFT	MutationTaster
*MAP3K7*	Chr:219919146‐219925643	c.149 T > C	p.Val50Ala	Deleterious (0.85)	Damaging	Deleterious

### 2.4. Plasmid Construction, Cell Culture, Transfection, and Western Blotting Analysis

Plasmids expressing Flag‐TAK1‐WT (WT), Flag‐TAK1‐Val50Ala (V50A), Flag‐TAK1‐Val50del (V50del), Flag‐TAK1‐Glu70Gln (E70Q) and Flag‐TAK1‐Pro512Leu (P512L) with the full coding sequence of *MAP3K7* (GenBank: NM_145331.3) were obtained from Miaoling Bioscience and Technology (Wuhan, China). All constructs were confirmed through sequencing. HEK293T cells were obtained from the American Type Culture Collection (Washington, DC, United States) and cultured at 37°C in a 5% CO_2_ atmosphere in Dulbecco′s modified Eagle medium supplemented with 10% fetal bovine serum. Subsequently, the cells were transfected with 2.5 *μ*g of plasmid using Lipofectamine 3000 (Thermo Fisher Scientific, Waltham, Massachusetts, United States) for 48 h before further use. An empty pCMV vector was used as the control. Transfected HEK293T cells were harvested and lysed in RIPA buffer supplemented with protease and phosphatase inhibitors. Transforming growth factor beta (TGF‐*β*) was added to the fresh culture medium at a concentration of 10 ng/mL for 24 h before collection. Protein concentrations were determined using a bicinchoninic acid protein assay kit. The proteins were then separated on 10% polyacrylamide gels and transferred to polyvinylidene fluoride membranes using electroblotting. Next, the membranes were blocked with 5% milk in Tris‐buffered saline containing Tween‐20 for 1 h and then incubated overnight at 4°C with primary antibodies in Table 3. The target proteins were detected using secondary goat antirabbit antibodies (diluted 1:8,000, BL003A, Biosharp, Hefei, China). Subsequently, the immunoblots were visualized using the ECL Plus Western blotting substrate and scanned with a Typhoon FLA 9500 (GE Healthcare Life Sciences, Fairfield, Michigan, United States).

**Table 3:** Primary antibodies used in this study.

Designation	Source	Identifiers	Dilution rate
Anti‐FLAG	Sigma‐Aldrich	F1804	1:1000
Anti‐TAK1	Cell Signaling Technology	5206S	1:1000
Anti‐Phospho‐TAK1(Thr187)	Cell Signaling Technology	4536S	1:1000
Anti‐GAPDH	Proteintech	10494‐1‐AP	1:10000
Anti‐p38	Proteintech	14064‐1‐AP	1:1000
Anti‐phospho‐p38(Thr180/Tyr182)	Cell Signaling Technology	4511 T	1:1000
Anti‐JNK	Proteintech	24164‐1‐AP	1:10000
Anti‐Phospho‐JNK (Tyr185)	Proteintech	80024‐1‐AP	1:2000
Anti‐NF‐*κ*B p65	Cell Signaling Technology	8242S	1:1000
Anti‐phospho‐NF‐*κ*B p65 (Ser536)	Cell Signaling Technology	3033S	1:2000

## 3. Statistical Analysis

Statistical analysis was conducted using GraphPad Prism 9.3 (San Diego, California, United States). The results are expressed as the mean ± standard deviation (SD). One‐way analysis of variance (ANOVA) was employed to assess statistical significance, which was indicated by *p* < 0.05.

## 4. Results

### 4.1. Clinical Manifestations

**Figure 2:**
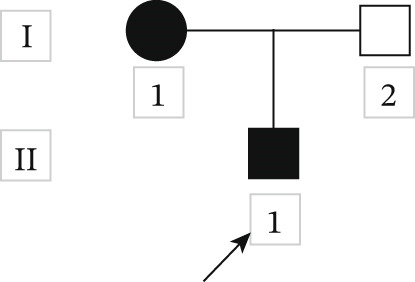
Pedigree of a Chinese family with autosomal dominant CSCF. Squares indicate males, circles indicate females. Solid black symbols indicate affected individuals. The proband is indicated with an arrow.

A small family presented with multiple congenital anomalies and developmental delays. Their pedigree chart is illustrated in Figure 2. The family included two individuals who were identified as affected (I‐1 and II‐1).

### 4.2. Proband (II‐1, Male, 47‐minute‐Old)

The proband, a 47‐minute‐old male, was the only child of nonconsanguineous Chinese parents. He was delivered by cesarean section at 36^+1^ weeks of gestation with manifestations of respiratory distress syndrome and low birth weight, which were potentially associated with maternal complications including cardiac disease and polyhydramnios. He had no history of asphyxia at birth.

**Figure 3:**
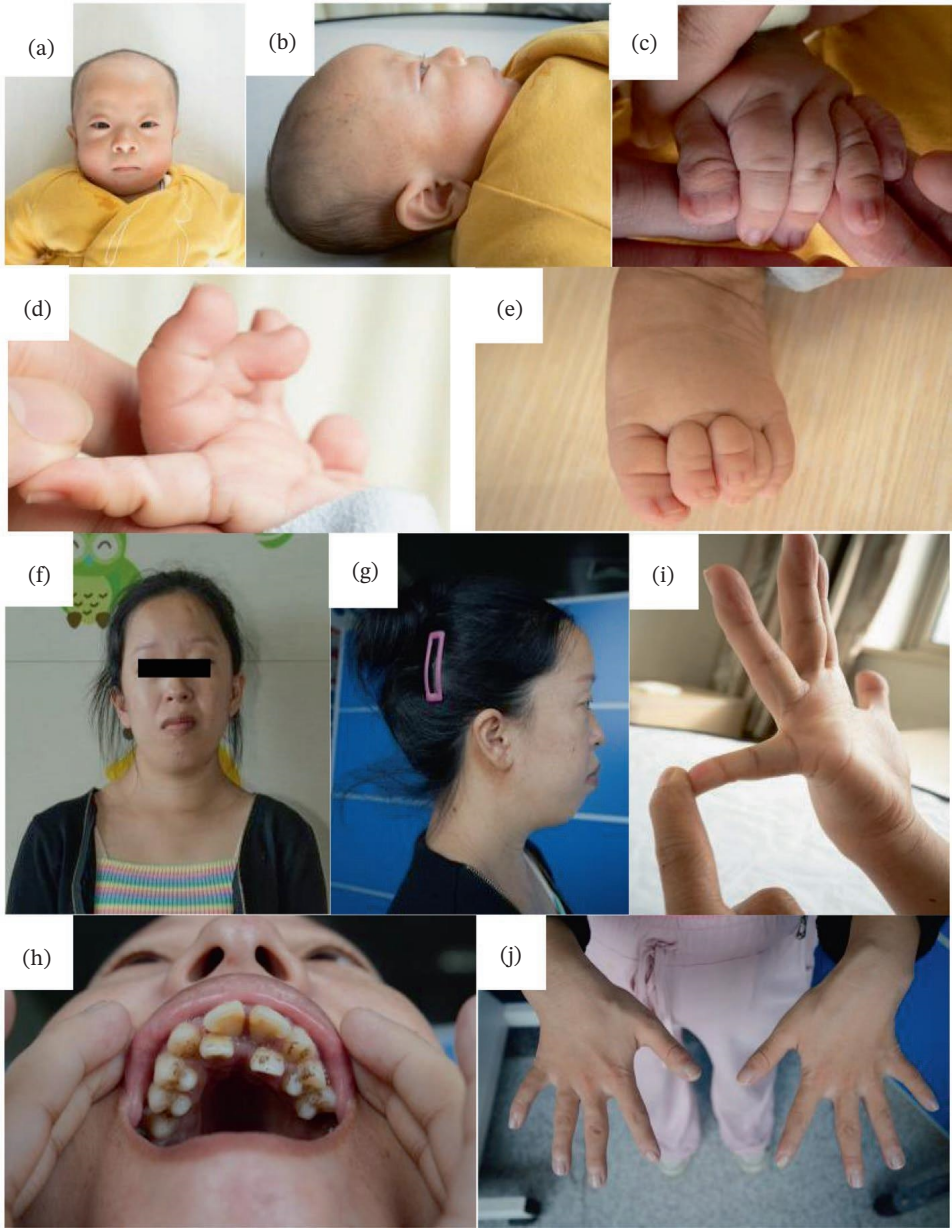
(a–i) Light photographs of patient and his mother with CSCF. (a–e) Patients show widely spaced eyes, low‐set ears, round‐tipped nose, anteverted nares, a long philtrum, full cheeks, syndactyly of the left thumb, bilateral overlapping toes, and hyperextensible fingers (f, g). The mother shows dental malocclusion, bilateral ptosis, hypertelorism, low‐set ears, broad nasal bridge, anteverted nares, long philtrum, dental malocclusion, and short and hyperextended fingers.

The proband presented with distinctive clinical features and a complex medical course. The infant exhibited characteristic craniofacial features, including hypertelorism, low‐set ears, broad nasal bridge, anteverted nares, a long philtrum, and full cheeks (Figure 3a,b). Notable findings included syndactyly of the left thumb, bilateral overlapping toes, hyperextensible fingers, and generalized hypotonia (Figures 3c, 3d, and 3e). The clinical course was complicated by recurrent respiratory distress requiring noninvasive ventilation, with subsequent diagnosis of neonatal pneumonia treated with cefoperazone for 7 days. Persistent stridor, attributed to congenital laryngomalacia, was observed during 4‐month follow‐up. Cardiac evaluation revealed patent ductus arteriosus, patent foramen ovale, and pulmonary hypertension, with subsequent identification of congenital bicuspid aortic valve. Feeding difficulties necessitated nasogastric support until 40^+4^ weeks corrected gestational age, when oral feeding was successfully established. During the physical examination at 1 year of age, his body weight was 7.08 kg (<−2 SD) and his height was 64.7 cm (<−2 SD), and head circumference was 45 cm.

### 4.3. Mother (I‐1, Female, 27 years Old)

**Figure 4:**
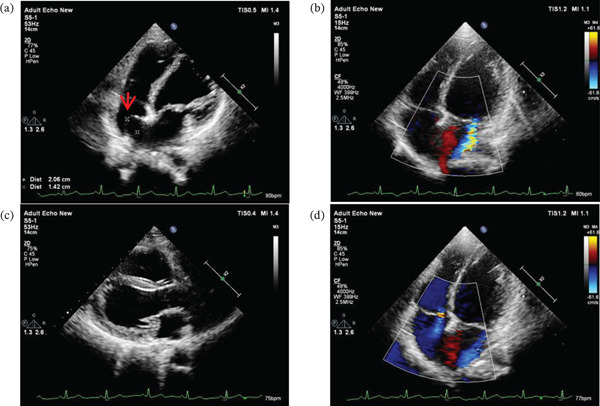
Echocardiogram of mother of proband. (a) Atrial septal aneurysm. (b) Thickened mitral valve with regurgitation. (c) Mitral valve prolapse. (d) Tricuspid regurgitation.

**Figure 5:**
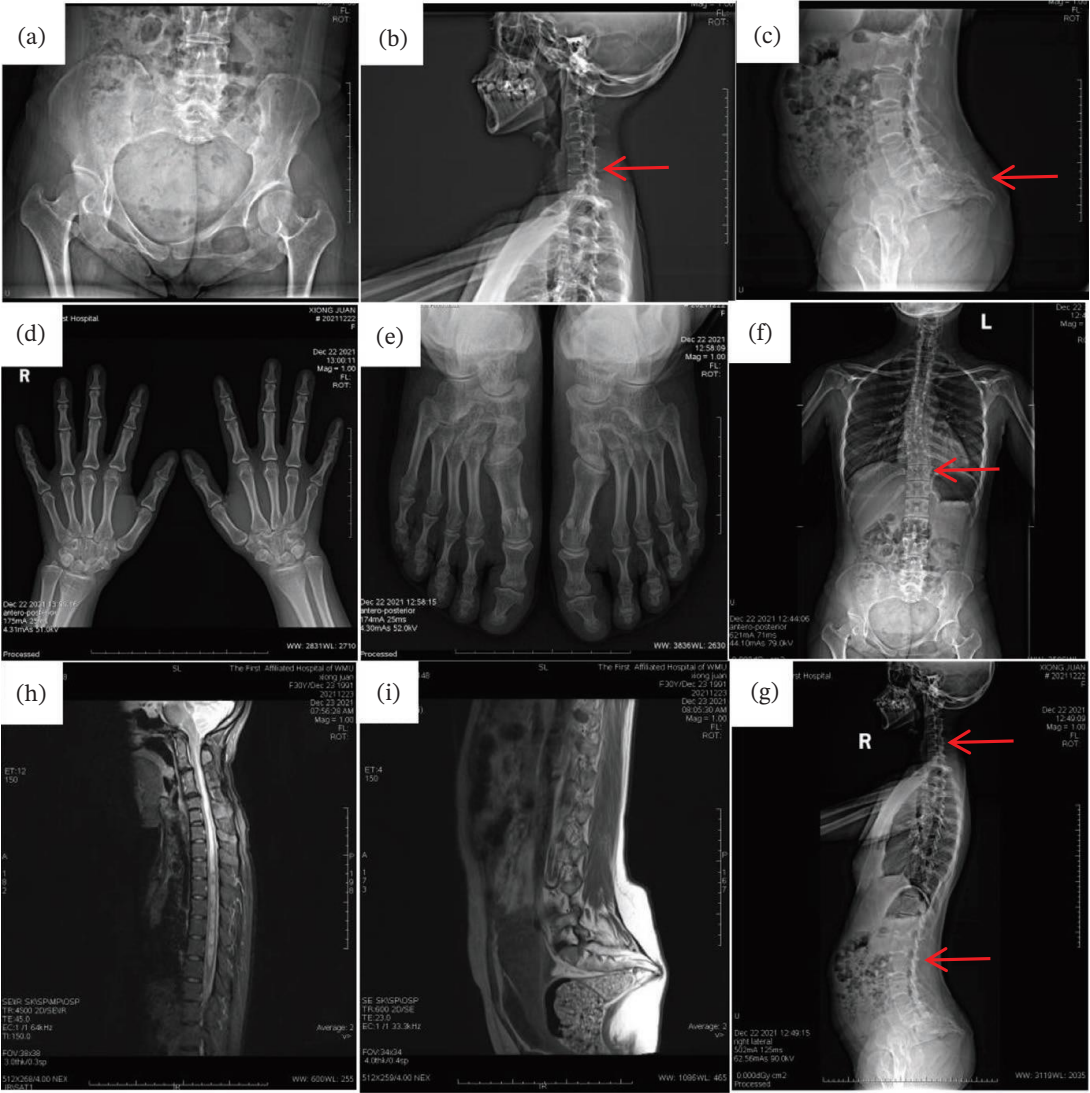
X‐ray and MRI of mother of patient. (a) Asymmetry hip. (b) Vertebral fusion at C2–3, C4–5, and C6–7 levels. (c) Horizontally oriented, hook‐shaped coccyx. (d, e) Absence of carpal and tarsal bone fusion. (f) thoracic scoliosis. (g) Loss of cervical lordosis with excessive lumbar lordosis. Spinal MRI show (h, i) heterogeneous signal intensity in thoracolumbar spinal cord with abnormal sacrococcygeal development, with a horizontally oriented, hook‐shaped coccyx.

The mother, a 27‐year‐old female with a height of 142 cm (<−2 SD), exhibited a constellation of clinical features including growth delay, distinctive facial characteristics (bilateral ptosis, hypertelorism, low‐set ears, broad nasal bridge, anteverted nares, long philtrum, and dental malocclusion) (Figures 3f, 3g, and 3h), and musculoskeletal abnormalities (short and hyperextended fingers and scoliosis) (Figure 3i,j). Comprehensive evaluation revealed significant cardiac valvular abnormalities including mitral valve prolapse with regurgitation, tricuspid regurgitation, and atrial septal aneurysm (Figure 4a, 4b, 4c, and 4d). Radiographic studies demonstrated multiple vertebral fusions (C2–3, C4–5, and C6–7), thoracolumbar spinal cord abnormalities suggestive of syringomyelia, and developmental anomalies of the sacrococcygeal region (Figures 5a, 5b, 5c, 5d, 5e, 5f, and 5g). Additional findings included conductive hearing loss, joint laxity, and a history of two miscarriages, one involving trisomy of Chromosome 4. This complex phenotype, presenting across two generations, suggests a potential genetic etiology warranting further investigation.

### 4.4. Genetic Findings

**Figure 6:**
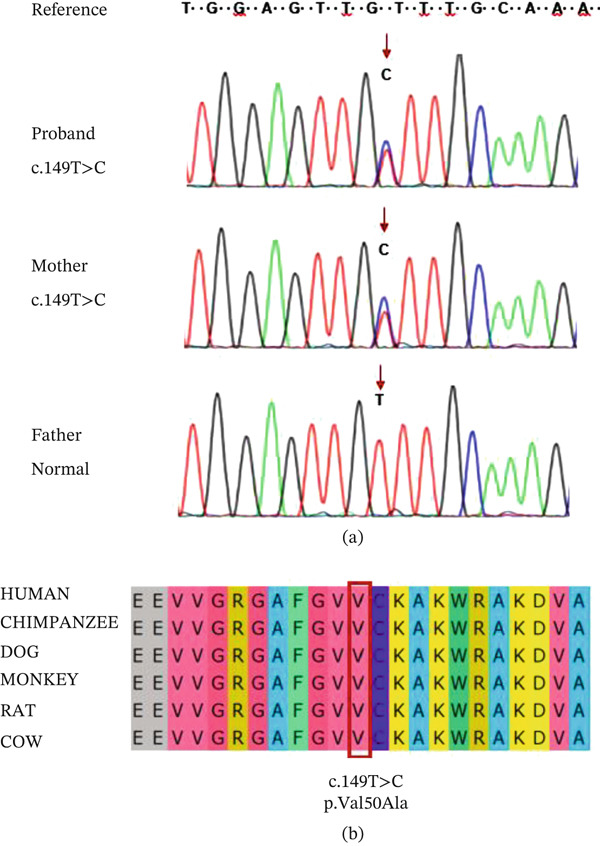
Sanger sequencing and conservative analysis. (a) Sanger sequencing showing the *M* *A* *P*3*K*7 c.149 T > C variant. The red arrows indicate variant sites. (b) Conservation of the p.Val50Ala variant across various species.

To identify potential variants responsible for these phenotypes in this family, we performed WES and Sanger sequencing on blood samples collected from the parents and proband, revealing a rare heterozygous variant in the *MAP3K7* gene in the proband. The variant was a single base substitution at coding sequence Positions 149 (NM_145331.3, c.149 T > C) in Exon 2 of *MAP3K7* in the proband and mother (Figure 6a). The father was not found to have the variant. This variant is predicted to result in the substitution of valine with alanine at position 50 (p.Val50Ala) in the Pkinase domain of the TAK1 protein (Figure 1b) and was classified as VUS (PM1 + PM2 + PP3) according to the ACMG guidelines [12]. Notably, this variant has not been reported previously in the 1000 Genomes Project, the Exome Aggregation Consortium, or the Genome Aggregation Database. These findings warranted further investigation into this variant.

### 4.5. P Assessment and Conservation Analysis

The pathogenicity of c.149 T > C in the *MAP3K7* gene was evaluated using several methods and was predicted to be deleterious (Table 2). This variant, c.149 T > C (p.Val50Ala), is located in the Pkinase domain of the TAK1 protein (Figure 1a), which is crucial for the phosphorylation activation of the TAK1 protein. Crucially, a comparison of *MAP3K7* sequences from various mammals, including human (UniProt: O43318), chimpanzee (UniProt: H2RCY2), dog (UniProt: A0A8C0MMM2), monkey (UniProt: A0A2K6UPI5), rat (UniProt: P0C8E4), and cow (UniProt: A2VDU3), revealed that amino acid 50 of TAK1 is 100% conserved across these species. Moreover, the conservation score of Val50 in PhastCons was 1, indicating that Val50 is highly conserved (Figure 6b). These findings suggest that Val50 plays a key role in the normal biological function of the TAK1 protein.

### 4.6. Effect on 3D Protein Structure

**Figure 7:**
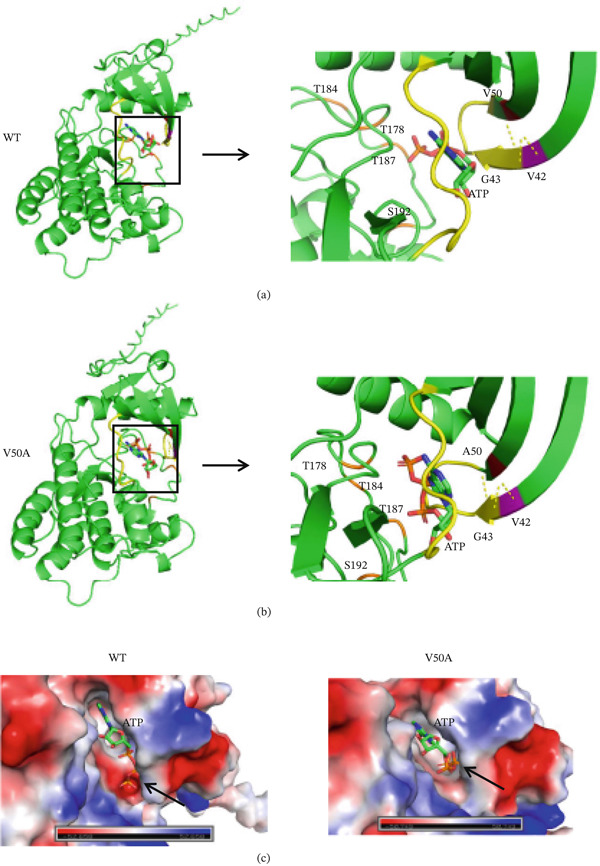
Crystal structure of the human TAK1‐ATP compound. (a) Location and hydrogen bonding of Val50 in the WT human TAK1‐ATP compound. (b) Location and hydrogen bonding of V50A in the mutant human TAK1‐ATP compound. (c) Schematic representation structure of human TAK1‐ATP compound. The structure of TAK1‐ATP compound was predicted using AlphaFold 3.0. WT: wild‐type. T: threonine, S: serine, G: glycine, A: alanine, V: valine, V50A: p.Val50Ala.

To investigate the effect of the variant on protein structure, we modeled the TAK1‐ATP complex structures of the V50A and wild‐type TAK1 proteins using AlphaFold 3.0. This valine residue at Position 50 was predicted to be part of the *β*‐sheet of the TAK1 protein and to form hydrogen bonds with V42 and G43 (Figure 7a,b). Although the hydrogen bonding pattern in the V50A mutant remains unchanged compared with the wild type, we found a significant change in the position of the phosphate groups of ATP within the ATP‐binding pocket, which may affect the phosphorylation of TAK1 (Figure 7c).

### 4.7. Functional Assessment of the Missense Variants Identified in *MAP3K7*

The clinical distinctions between FMD2 and CSCF patients are believed to be caused by distinct molecular pathways. FMD2 is generally considered to be gain‐of‐function variants, whereas CSCF is loss‐of‐function variants of *MAP3K7.* However, the impact of these variants on function is still unknown. In our study, in combination with already published patients, we analyzed HEK293T cells transfected with expression plasmids encoding FLAG‐tagged wild‐type (WT), TAK1p.Val50Ala (V50A), TAK1p.Val50del (V50del), TAK1 p.Glu70Gln (E70Q), and TAK1p.Pro512Leu (P512L).

**Figure 8:**
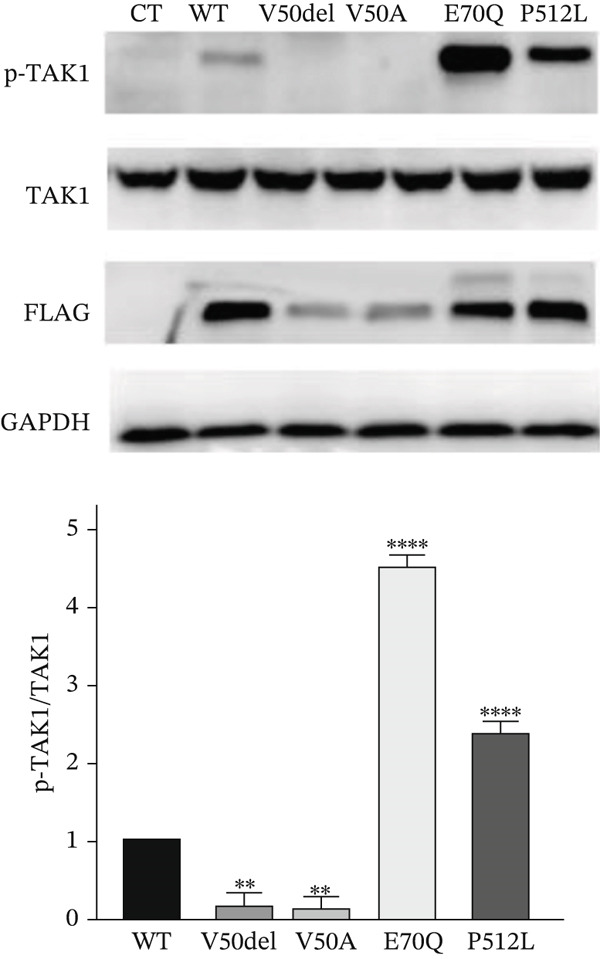
Differential expression and autophosphorylation of variants in *MAP3K7* causing CSCF or FMD2. Western blot analysis shows reduced expression levels of phospho‐TAK1(Thr187) in V50del and V50A variants of CSCF compared with WT and increased expression levels in E70Q and P512L of FMD2, when overexpressed in HEK293T cells. Cells transfected with an empty vector were used as control. All data are shown as the mean ± SD of three independent experiments. Statistically significant differences are denoted by asterisks ( ^∗^) with a significance level of  ^∗∗^ *p* < 0.01 and  ^∗∗∗∗^ *p* < 0.0001. CT: control.

Western blotting analysis of the whole‐cell extracts showed that V50A and V50del exhibited significantly reduced phosphorylation at Thr187 compared with WT, whereas E70Q and P512L displayed markedly enhanced Thr187 phosphorylation relative to WT (Figure 8). Notably, the flag‐tagged protein showed a significant decrease in CSCF variants, suggesting that these variants might affect TAK protein stability.

**Figure 9:**
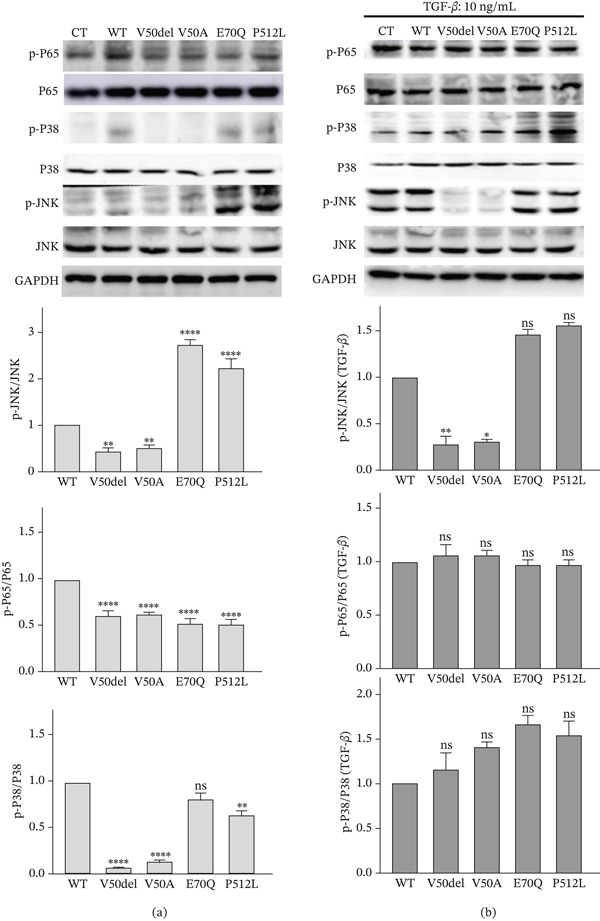
Western blot analysis of MAP3K7 downstream targets in HEK293T cells under basal and TGF‐*β*–stimulated conditions. (a) Basal condition: V50del, V50A, E70Q, and P512L variants exhibit reduced phospho‐NF‐*κ*B p65 (Ser536) and phospho‐p38 (Thr180/Tyr182) levels relative to WT; E70Q does not follow the trend for p‐p38. Phospho‐JNK (Tyr185) is decreased in V50del/V50A and increased in E70Q/P512L, consistent with phospho‐TAK1 (Thr187). (b) TGF‐*β*–stimulated condition, 10 ng/mL, 24 h: TGF‐*β* stimulation restores phospho‐p65 and phospho‐p38 signals in all mutants to WT‐like levels, whereas phospho‐JNK shows no significant trend changes. All data are shown as the mean ± SD of three independent experiments. Statistically significant differences are denoted by asterisks ( ^∗^) with a significance level of  ^∗^ *p* < 0.05 and  ^∗∗^ *p* < 0.01. CT: control.

Given the complex clinical manifestations observed in patients, we focused on three well‐known downstream molecules of TAK1: p65, p38, and JNK. Western blot analysis revealed that among four variants, the expression levels of pp38 and pp65 were both decreased, with the E70Q variant showing no significant reduction in pp38 (Figure 9a). Surprisingly, upon stimulation with TGF‐*β*, both pp38 and pp65 rebounded to levels comparable to the WT, suggesting the potential therapeutic utility of TGF‐*β* (Figure 9b). In addition, the consistent trend of change observed in p‐JNK aligns with that of p‐TAK1, and the lack of a significant rebound in p‐JNK levels following TGF‐*β* stimulation suggests that additional pathways might be involved in the regulation of p‐JNK. These findings indicate that the stability and autophosphorylation of TAK1 at Thr187 could serve as a potential molecular signature to differentiate between FMD2 and CSCF variants, and not all downstream pathways of TAK1 are necessarily differentially affected, which is consistent with the research by van Woerden et al. [6]. In addition, our innovative use of TGF‐*β* stimulation has, to some extent, altered the expression of downstream molecules, offering new possibilities for molecular therapeutic interventions.

## 5. Discussion

In this study, we identified a family with CSCF due to a novel *M* *A* *P*3*K*7 c.149 T > C variant and assessed the genotype–phenotype correlation for *MAP3K7* variants, expanding the understanding of the clinical characteristics associated with this gene. Further functional analysis indicated that TGF‐*β* can partially reverse the changes in downstream molecules, thus offering possibilities for clinical treatment.

Our study provides the first comprehensive summary of patients with CSCF and FMD2 associated with *MAP3K7* variants, encompassing 24 CSCF patient families (22 variants) and 22 FMD2 patient families (5 variants) (File S1). All variants are located in the domains of the TAK1 protein. Notably, the variants of CSCF patients are all within the kinase domain, whereas the variants of FMD2 patients can also be located in the TAB2/3 binding domain. Interestingly, FMD2 patients have a significant hot variant, highlighting the essential role of proline at Position 512 in disease pathogenesis. They share facial characteristics such as full cheeks, hypertelorism, palpebral fissures, peri/supraorbital fullness, round‐tipped nose, and micrognathia. In addition, CSCF patients have some unique facial features, including low‐set ears, posteriorly rotated ears, and long philtrum. These facial features will assist doctors in making a differential diagnosis during the initial consultation. Congenital heart disease seems to be common in patients with *MAP3K7* variants, with valvular abnormalities being more common than septal defects and cardiomyopathies, which may be related to the key role of TAK1 in vascular development [14, 15]. Shared skeletal features include scoliosis, vertebral fusion, carpal fusion, tarsal fusion, and brachydactyly. Keloid and flexion contractures are characteristic of FMD2 patients, whereas CSCF patients more often exhibit joint laxity. Besides, intellectual disability occurs in both groups of patients, with a higher incidence in FMD2 patients, and these patients do not show significant changes in brain MRI, suggesting that the disorder may be unrelated to structural changes. In addition, hearing loss seems to be a common manifestation in all patients, coupled with morphological changes in the external and internal ear, indicating the important role of TAK1 in ear development. CSCF patients seem more prone to digestive and urinary system disorders. Moreover, cryptorchidism is also highly prevalent in both types of patients (Table 1). It can be seen that the clinical manifestations of patients with *MAP3K7* variants are systemic, which poses a great challenge to clinical treatment. In the clinical manifestations of our V50A variant patients, low‐set ears, long philtrum, hypotonia, feeding difficulties, and the absence of flexion contractures suggest the possibility of being a CSCF patient, and our functional experiments can substantiate this.

In our study, we evaluated the changes in Thr187 phosphorylation for the V50A, V50del, E70Q, and P512L variants. Consistent with previous research, CSCF is a loss‐of‐function variant, whereas FMD2 is a gain‐of‐function variant. In addition, we further explored downstream molecules and found that p‐JNK changes in concert with p‐TAK1, suggesting that p‐TAK1 may have a more direct role in regulating p‐JNK. On the other hand, p‐P38 and p‐P65 showed a consistent decrease after the variant, which may be related to the high clinical overlap between CSCF and FMD2 patients. We further stimulated with TGF‐*β* and found that p‐P38 and p‐P65 could be restored, but p‐JNK did not show a significant recovery, further indicating that p‐JNK may be directly regulated by p‐TAK1. In summary, our functional studies provide insights into the correlation between clinical phenotypes and genotypes, offering possibilities for clinical treatment.

Here, we conducted a further description of the genotype–phenotype correlation, but due to the limited number of cases and descriptive differences, our table is not perfect. Besides, due to sample limitations, we only conducted wild‐type and mutant plasmid overexpression experiments in HEK293T cells. The extraction and analysis of cell cultures from patients or the transfection of plasmids into semiknockdown cell lines would be more effective in simulating the in vivo environment, producing results closer to those in the internal environment. However, based on the clinical presentation of the patient and our analysis, we believe that this did not affect our results.

In conclusion, we identified a new heterozygous loss‐of‐function variant, *c*.149 *T* > *C* (p.Val50Ala), in *MAP3K7* that causes CSCF. Besides, we evaluated the genotype–phenotype correlation for *MAP3K7* variants, enhancing the comprehension of the clinical features associated with this gene. Further, functional analysis reveals that TGF‐*β* can partially counteract the alterations in downstream molecules, suggesting potential avenues for clinical therapy. These findings contribute to our understanding of the molecular pathogenesis of *MAP3K7* variants and the clinical identification and intervention of *MAP3K7* variants.

## Author Contributions

Conceptualization and design: Dan Wang, and Jia Li. Acquisition of data: Ting Zhou, Jiamin Shi, Tingmin Zhou, Jia Li, Xinru Fu, Fengzhen Xu, Chuangjie Gu, Danping Wang, and Ruiting Wu. Analysis and interpretation of data: Ting Zhou, Jia Li, and Tingmin Zhou . Drafting the article: Ting Zhou and Jiamin Shi. Supervision: Danping Wang, Jingling Shen, and Li Liu. All the authors contributed to revising the manuscript and reading the manuscript.

## Funding

This study was supported by the National Natural Science Foundation of China (82171701), the Social Programs of Wenzhou Technology Bureau (Y2023004), and Consortium for infection and Innovation (CII), The First Affiliated Hospital of Wenzhou Medical University (2025WMU‐X001).

## Disclosure

All the authors approved the submitted version.

## Ethics Statement

The proband and family members were enrolled at the First Affiliated Hospital of Wenzhou Medical University in May 2022. Written informed consent was obtained from the family of the proband before the beginning of the study. Our study was approved by the Ethics Committee of the First Affiliated Hospital of Wenzhou Medical University (Ethics Approval Number KY2022‐R177).

## Consent

Written informed consent was obtained for the participation. The participants have consented to have their data published.

## Conflicts of Interest

The authors declare no conflicts of interests.

## Supporting information


**Supporting Information** Additional supporting information can be found online in the Supporting Information section. File S1. The online version contains supporting excel available at Supporting Information for review and publication.

## Data Availability

The data supporting the findings of this study are available from the corresponding authors upon reasonable request.
